# 15-year incidence of new-onset diabetic ketoacidosis in children with type 1 diabetes from a regional paediatric setting (Auckland, New Zealand)

**DOI:** 10.1186/1687-9856-2015-S1-P3

**Published:** 2015-04-28

**Authors:** Craig Jefferies, Samuel Cutfield, José GB Derraik, Jignal Bhagvandas, Benjamin B Albert, Paul L Hofman, Alistair J Gunn, Wayne S Cutfield

**Affiliations:** 1 Starship Children’s Hospital, Auckland, New Zealand; 2 Liggins Institute, University of Auckland, Auckland, New Zealand; 3 Department of Physiology, University of Auckland, Auckland, New Zealand; 4 Gravida National centre for growth and development, Auckland, New Zealand

## Aims

This study aimed to assess the incidence of new-onset diabetic ketoacidosis (DKA) incidence over a 15-year period in children with type 1 diabetes (T1DM) from our regional paediatric diabetes centre in Auckland, New Zealand.

## Methods

We performed a retrospective review of all patients <15 years of age diagnosed with T1DM from our unselected complete regional cohort for the years 1999 to 2013. Children were included if they had type 1 diabetes as defined by clinical presentation and/or DKA and/or presence of pre-type 1 diabetes autoantibodies. DKA was classified into Mild (pH <7.3 bicarbonate <15), Mod (pH <7.2 bicarbonate <10), and severe (pH <7.1 bicarbonate <5), according to the ISPAD guidelines.

## Results

For the 15-year period, there were 731 children <15 years with new-onset T1DM, 343 (47%) males, there were 195 (26.7%) cases of new-onset DKA: 51 (26%) severe, 52 (27%) moderate, and 92 (47%) with mild DKA. Average age at diagnosis was 8.6 years. The annual incidence of DKA was variable, ranging from 18% to 37%. The overall incidence of new-onset DKA did not differ over the study interval (p=0.78). The likelihood of being in DKA at onset of T1DM was unaffected by age, sex, ethnicity, or socio-economic status. Amongst those in DKA, New Zealand European ethnicity (p=0.038) and female gender (p=0.056) were each associated with increasing DKA severity at presentation.

## Conclusions

There has been a stable but persistent level of New-onset DKA over the 15-year period studied in our regional paediatric population. Action must be taken to improve awareness of T1DM and in doing so, reduce the incidence of new-onset DKA.

